# Supplementary material for: Microencapsulation of Camellia oleifera Seed Oil Emulsion By-Products: Structural Characterization and Lipidomics Analysis
Source: Foods. 2025 Sep 24;14(19):3314. doi: 10.3390/foods14193314 (PMC12523875; doi:10.3390/foods14193314)
Supplement: Supplementary file 1 [file foods-14-03314-s001.zip › Supplementary Table.pdf]

Supplementary table 1: lipid composition analysis table of microcapsules

Compounds	Class I	Class II	OPM	EPM
Cer(d18:0/22:1(2OH))	SP	Cer	0.7651±0.13	1.3955±0.17
Cer(d18:2/22:1)	SP	Cer	0.2088±0.03	0.1496±0.04
Cer(d18:2/16:1)	SP	Cer	12.0237±1.91	10.3321±1.26
Cer(d18:2/18:1)	SP	Cer	N/A	0.1516±0.03
Cer(t18:0/18:0(2OH))	SP	Cert	3.8138±1.10	3.8473±0.36
Cer(t18:0/22:0(2OH))	SP	Cert	0.1788±0.08	0.1601±0.06
Cer(t18:0/24:0(2OH))	SP	Cert	0.6638±0.07	0.7171±0.21
Cer(t18:0/25:0(2OH))	SP	Cert	0.2139±0.02	N/A
Cer(t18:0/26:0(2OH))	SP	Cert	0.1650±0.10	N/A
Cer(t18:1/16:0(2OH))	SP	Cert	0.4188±0.15	0.2992±0.13
Cer(t18:1/26:0)	SP	Cert	0.3567±0.17	0.5425±0.09
Cer(t18:1/23:0(2OH))	SP	Cert	0.3530±0.04	0.3570±0.09
Cer(t18:1/25:0(2OH))	SP	Cert	0.7614±0.34	0.7324±0.18
Cer(t18:1/26:0(2OH))	SP	Cert	0.9012±0.02	0.7730±0.21
Cer(t18:0/22:0)	SP	Cert	0.2837±0.03	0.2232±0.05
Cer(t18:0/24:0)	SP	Cert	0.4314±0.12	0.7196±0.36
Cer(t18:0/26:0)	SP	Cert	N/A	0.2805±0.08
Cer(t18:1/24:0)	SP	Cert	3.1185±0.26	3.5063±0.23
Cer(t18:1/22:0(2OH))	SP	Cert	7.6269±0.95	7.1082±0.15
Coenzyme Q10	PR	CoQ	3.7075±1.07	5.1534±1.03
Coenzyme Q8	PR	CoQ	0.1205±0.02	0.2582±0.05
Coenzyme Q9	PR	CoQ	0.7574±0.23	1.2267±0.16
DG (16:1_18:2)	GL	DG	3.4948±0.33	7.7421±0.45
DG (16:0_18:0)	GL	DG	165.5691±4.88	170.0559±23.01
DG (14:0_18:0)	GL	DG	1.2108±0.23	0.9646±0.12
DG (16:0_16:0)	GL	DG	58.8331±8.68	67.3591±4.73
DG (14:0_16:0)	GL	DG	0.8137±0.36	1.4819±0.32
DG (16:1_20:2)	GL	DG	2.5068±0.25	5.5728±0.99
DG (18:1_18:2)	GL	DG	559.9819±31.25	1367.7995±187.92
DG (14:1_22:2)	GL	DG	2.2081±0.32	5.1080±1.38
DG (18:0_18:3)	GL	DG	0.6738±0.18	1.8242±0.46
DG (19:1_18:2)	GL	DG	0.4567±0.05	0.7147±0.14
DG (18:2_22:1)	GL	DG	1.1973±0.31	2.4027±0.41
DG (14:0_20:0)	GL	DG	1.6087±0.22	1.8811±0.64
DG (16:0_20:4)	GL	DG	0.6077±0.11	0.9806±0.12
DG (18:2_18:2)	GL	DG	545.4760±11.38	837.5426±83.70
DG (16:1_20:3)	GL	DG	2.7360±0.44	4.3801±1.00
DG (18:1_18:3)	GL	DG	54.5655±3.34	99.1740±9.66
DG (18:2_20:2)	GL	DG	0.2625±0.04	0.4281±0.08
DG (18:2_18:3)	GL	DG	16.9722±1.87	27.5979±2.41
DG (18:3_18:3)	GL	DG	1.7369±0.40	1.6696±0.07

DG (16:4_18:3)	GL	DG	0.2605±0.04	N/A
DG (18:2_20:1)	GL	DG	5.1285±0.88	13.1255±1.03
DG (17:0_18:0)	GL	DG	0.4618±0.12	0.6237±0.25
DG (18:0_18:0)	GL	DG	97.1960±8.46	108.9153±16.42
DG (16:0_20:0)	GL	DG	1.8515±0.42	2.0985±0.25
DG (18:2_25:0)	GL	DG	0.4734±0.10	0.7435±0.17
DG (18:2_24:0)	GL	DG	4.0123±0.56	4.1593±0.76
DG (18:2_23:0)	GL	DG	0.6618±0.04	0.7085±0.10
DG (18:2_22:0)	GL	DG	2.2046±0.51	3.0064±0.42
DG (18:1_20:1)	GL	DG	15.9390±2.47	72.8134±6.73
DG (18:1_18:1)	GL	DG	1256.3904±108.60	2885.7161±946.10
DG (18:0_18:2)	GL	DG	29.4184±2.93	67.6124±7.69
DG (17:0_18:2)	GL	DG	0.9105±0.21	1.5695±0.31
DG (17:1_18:1)	GL	DG	1.0858±0.20	3.0067±0.56
DG (16:0_18:2)	GL	DG	282.2599±17.50	480.2897±53.07
DG (16:0_18:3)	GL	DG	5.9676±0.56	9.3608±1.20
DG (16:1_18:1)	GL	DG	1.0804±0.68	5.0696±1.91
DG (14:0_18:2)	GL	DG	1.0114±0.21	2.8917±0.39
DG (16:2_18:1)	GL	DG	N/A	0.2554±0.10
DG (16:0_20:1)	GL	DG	0.6251±0.04	2.8541±0.85
DG (17:0_18:1)	GL	DG	1.5558±0.20	2.5933±0.44
DG (16:0_18:1)	GL	DG	233.4675±13.55	542.1331±77.35
DG (16:0_16:1)	GL	DG	0.3115±0.11	0.4776±0.19
DG (14:0_18:1)	GL	DG	0.6836±0.13	2.6150±0.13
DG (14:0_16:1)	GL	DG	3.0207±0.38	1.9448±1.54
DG (20:0_18:0)	GL	DG	1.1381±0.22	1.1734±0.25
DG (14:0_22:0)	GL	DG	1.8408±0.54	2.0752±0.23
DG (15:0_18:2)	GL	DG	0.2057±0.06	0.3598±0.02
DG (18:0_18:1)	GL	DG	63.6153±10.31	366.1075±29.03
DG (18:2_18:4)	GL	DG	0.2352±0.17	0.3855±0.12
DGCC (14:0_16:1)	GL	DGCC	1.0020±0.67	N/A
DGDG (16:0_18:3)	GL	DGDG	8.9932±2.17	10.3631±2.00
DGDG (16:0_16:0)	GL	DGDG	0.7876±0.13	0.7408±0.38
DGDG (16:0_18:1)	GL	DGDG	5.3482±1.31	11.4418±1.72
DGDG (16:0_18:2)	GL	DGDG	21.4177±1.62	28.1246±1.62
DGDG (18:0_18:2)	GL	DGDG	8.8849±0.59	12.3528±1.28
DGDG (18:0_18:3)	GL	DGDG	3.2495±0.72	3.7165±0.67
DGDG (18:1_18:2)	GL	DGDG	2.8118±0.96	8.3807±0.27
DGDG (18:1_18:3)	GL	DGDG	4.8552±0.42	6.3101±2.27
DGDG (18:2_18:2)	GL	DGDG	16.5198±3.35	23.8381±1.50
DGDG (14:0_20:5)	GL	DGDG	0.7217±0.16	1.1180±0.34
DGDG (18:2_18:3)	GL	DGDG	9.0383±0.60	8.4073±2.44
DGDG (18:3_18:3)	GL	DGDG	14.5415±1.38	15.8897±2.09
DGGA (16:0_18:2)	GL	DGGA	9.8381±1.41	11.6286±0.62

DGTS (18:2_20:5)	GL	DGTS	91.6409±13.89	95.5866±12.57
DGTS (18:1_20:5)	GL	DGTS	8.6323±1.96	12.9957±1.39
DGTS (18:3_20:5)	GL	DGTS	7.7927±1.60	6.0639±2.26
DGTS (18:3_18:3)	GL	DGTS	4.5755±1.82	3.9601±0.85
DGTS (18:1_20:4)	GL	DGTS	2.1346±0.18	9.5577±2.58
DGTS (18:2_18:3)	GL	DGTS	77.9655±4.46	86.9738±12.92
DGTS (16:0_20:5)	GL	DGTS	17.6362±2.65	18.4487±3.52
DGTS (16:0_18:2)	GL	DGTS	N/A	1.4138±0.41
DGTS (14:0_18:4)	GL	DGTS	2.4660±0.52	3.3906±0.55
DGTS (18:2_18:2)	GL	DGTS	4.5514±0.85	4.6387±0.23
DGTS (16:0_18:1)	GL	DGTS	N/A	2.0642±0.96
FFA (16:1)	FA	FFA	N/A	5.7046±0.82
FFA (35:0)	FA	FFA	23.2168±2.40	21.4029±4.29
FFA (28:0)	FA	FFA	14.0350±1.04	30.7987±13.54
FFA (24:0)	FA	FFA	1.3556±0.12	1.8069±0.32
FFA (22:0)	FA	FFA	2.5735±1.25	2.7578±0.42
FFA (21:0)	FA	FFA	1.3234±0.90	N/A
FFA (10:0)	FA	FFA	37.1439±6.93	29.7743±0.70
FFA (18:1)	FA	FFA	422.5327±21.90	2282.7040±341.91
FFA (20:0)	FA	FFA	1.3551±0.44	N/A
FFA (19:1)	FA	FFA	N/A	3.3635±0.92
FFA (20:1)	FA	FFA	5.3021±1.46	24.2087±8.02
FFA (22:1)	FA	FFA	3.2235±0.62	4.8808±1.46
FFA (24:1)	FA	FFA	N/A	3.1813±0.39
FFA (18:2)	FA	FFA	298.8083±56.45	711.8758±54.89
FFA (18:3)	FA	FFA	7.9770±1.01	12.2426±1.20
FFA (22:3)	FA	FFA	N/A	0.5676±0.14
FFA (17:1)	FA	FFA	0.8172±0.27	4.2220±0.18
FFA (18:0)	FA	FFA	307.1824±33.86	295.8196±27.14
FFA (17:0)	FA	FFA	3.0508±0.91	4.9438±0.38
FFA (15:0)	FA	FFA	1.7036±0.27	1.7180±0.32
FFA (14:0)	FA	FFA	3.5012±0.24	3.5789±0.13
FFA (16:0)	FA	FFA	415.2196±76.54	499.9515±28.78
HexCer(d18:2/24:0)	SP	HexCer	0.1839±0.02	0.1452±0.01
HexCer(d18:1/16:1)	SP	HexCer	1.7143±0.25	1.2801±0.13
HexCer(d16:1/18:0)	SP	HexCer	0.0852±0.05	0.0825±0.05
HexCer(t18:1/24:0(2OH))	SP	HexCer	0.2360±0.04	0.3333±0.06
HexCer(t18:1/22:0(2OH))	SP	HexCer	0.7960±0.12	0.7757±0.22
HexCer(d18:2/24:1)	SP	HexCer	0.8872±0.11	0.9480±0.09
LDGTS (16:0)	GL	LDGTS	N/A	0.6270±0.06
LDGTS (18:1)	GL	LDGTS	N/A	0.1681±0.09
LPA (18:3)	GP	LPA	13.0892±6.54	6.2055±0.96
LPA (22:5)	GP	LPA	2.0457±0.70	1.2142±0.08
LPA (18:2)	GP	LPA	164.1100±65.06	87.6986±8.41

LPA (18:0)	GP	LPA	16.3747±4.31	10.7735±0.79
LPA (16:0)	GP	LPA	62.0798±19.72	36.2830±2.73
LPA (18:1)	GP	LPA	19.5441±3.76	18.9890±1.72
LPC (22:0)	GP	LPC	1.2568±0.38	0.8829±0.08
LPC (20:0)	GP	LPC	0.9217±0.27	0.9013±0.11
LPC (19:0)	GP	LPC	0.0550±0.01	N/A
LPC (18:0)	GP	LPC	24.7213±4.70	18.8329±0.29
LPC (16:0)	GP	LPC	928.2866±66.96	792.4664±40.24
LPC (24:0)	GP	LPC	0.4001±0.12	0.3914±0.14
LPC (15:0)	GP	LPC	2.1844±0.24	1.7010±0.23
LPC (14:0)	GP	LPC	4.6192±0.68	3.6608±0.25
LPC (16:3)	GP	LPC	0.4959±0.34	0.2033±0.04
LPC (20:2)	GP	LPC	0.3009±0.04	0.2246±0.01
LPC (18:2)	GP	LPC	302.6990±27.81	354.7770±1.70
LPC (17:0)	GP	LPC	3.3650±0.91	2.4754±0.23
LPC (15:1)	GP	LPC	2.2874±0.26	1.8046±0.06
LPC (18:1)	GP	LPC	112.2855±1.88	115.9161±3.22
LPC (17:1)	GP	LPC	1.0134±0.20	0.7341±0.07
LPC (19:1)	GP	LPC	0.3349±0.05	0.2996±0.04
LPC (18:4)	GP	LPC	1.3083±0.58	0.4643±0.10
LPC (18:3)	GP	LPC	78.4353±37.21	37.3558±3.84
LPC (20:1)	GP	LPC	0.5110±0.06	0.5477±0.03
LPC (24:1)	GP	LPC	0.5445±0.04	0.7122±0.07
LPC (16:1)	GP	LPC	5.0386±0.17	4.8459±0.18
LPE (18:3)	GP	LPE	3.4581±2.34	1.6319±0.10
LPE (18:2)	GP	LPE	15.5059±2.23	12.8858±1.27
LPE (16:1)	GP	LPE	0.2061±0.07	N/A
LPE (22:0)	GP	LPE	0.8155±0.17	0.5853±0.21
LPE (20:0)	GP	LPE	0.3308±0.13	0.2073±0.14
LPE (18:0)	GP	LPE	2.8425±0.88	1.9601±0.12
LPE (16:0)	GP	LPE	35.8348±6.17	27.1022±0.82
LPE (18:1)	GP	LPE	2.6180±0.40	3.0109±0.17
LPG (16:0)	GP	LPG	18.9942±7.45	11.1721±0.61
LPG (18:0)	GP	LPG	2.2789±1.06	1.1197±0.11
LPG (18:1)	GP	LPG	1.4090±0.85	0.9639±0.03
LPG (18:2)	GP	LPG	7.4425±11.55	1.5399±0.23
LPI (18:2)	GP	LPI	291.1479±230.45	68.7017±3.36
LPI (18:0)	GP	LPI	97.2060±54.53	33.4549±3.97
LPI (18:1)	GP	LPI	23.4785±16.63	14.7397±2.43
LPI (16:0)	GP	LPI	278.1174±181.43	92.7244±9.57
MG (16:0)	GL	MG	416.4692±41.84	805.1635±142.82
MG (18:3)	GL	MG	1.3549±0.42	3.4439±0.59
MG (18:2)	GL	MG	12.9335±2.56	68.1271±13.33
MG (18:1)	GL	MG	38.0869±8.39	338.0275±63.17

MG (14:0)	GL	MG	3.8121±0.71	8.3540±1.69
MG (18:0)	GL	MG	292.0553±36.94	607.6349±106.35
MGDG (18:1_18:2)	GL	MGDG	1.4534±0.41	N/A
MGDG (18:2_18:3)	GL	MGDG	1.6672±0.97	N/A
MGDG (18:2_18:4)	GL	MGDG	2.0345±0.61	N/A
MGDG (18:3_18:3)	GL	MGDG	4.9054±1.40	4.7728±2.38
MGDG (16:0_18:2)	GL	MGDG	2.5149±0.65	2.7264±0.75
MGDG (18:2_18:2)	GL	MGDG	6.5887±1.67	7.9677±1.12
PA (18:2_18:3)	GP	PA	58.3848±22.02	57.7329±28.02
PA (18:1_18:3)	GP	PA	5.4745±2.26	6.0292±2.50
PC (16:0_16:0)	GP	PC	18.3193±2.07	13.1149±0.29
PC (18:0_14:0)	GP	PC	3.1617±0.63	2.7218±0.44
PC (16:0_18:0)	GP	PC	1.2856±0.33	0.8663±0.09
PC (16:0_16:1)	GP	PC	3.0615±0.65	1.9122±0.72
PC (14:0_18:1)	GP	PC	8.6196±1.78	7.2118±0.53
PC (16:0_18:1)	GP	PC	63.5350±8.80	77.4121±2.32
PC (18:1_22:0)	GP	PC	0.2899±0.04	0.2354±0.07
PC (14:0_18:2)	GP	PC	4.4645±1.15	3.0033±0.33
PC (15:0_18:2)	GP	PC	2.9360±1.04	1.8098±0.50
PC (16:1_18:1)	GP	PC	N/A	0.9091±0.09
PC (16:0_18:2)	GP	PC	575.5218±65.49	424.7577±29.58
PC (17:1_18:1)	GP	PC	1.3889±0.18	1.4450±0.31
PC (17:0_18:2)	GP	PC	5.6017±0.48	3.6713±0.89
PC (18:1_18:1)	GP	PC	11.8322±1.82	42.7007±2.24
PC (24:0_18:2)	GP	PC	1.3919±0.14	1.1995±0.15
PC (16:0_18:3)	GP	PC	31.6805±7.04	24.1261±2.48
PC (16:1_18:2)	GP	PC	2.6940±1.29	2.3502±0.27
PC (17:1_18:2)	GP	PC	7.0223±1.05	6.6286±0.28
PC (18:1_18:2)	GP	PC	118.9533±18.47	113.1278±10.62
PC (18:2_18:2)	GP	PC	378.4557±60.39	296.0828±21.36
PC (18:3_18:1)	GP	PC	8.7041±2.07	6.7256±0.75
PC (18:2_16:3)	GP	PC	0.7385±0.42	N/A
PC (18:0_18:3)	GP	PC	11.5053±1.34	7.2507±0.55
PC (18:2_18:3)	GP	PC	62.5139±7.52	43.0442±1.37
PC (18:3_18:3)	GP	PC	9.0496±1.31	6.9788±1.52
PC (18:0_12:0)	GP	PC	0.4320±0.10	N/A
PC (18:0_18:2)	GP	PC	141.9975±15.45	94.6280±8.03
PC (20:1_18:2)	GP	PC	1.9739±0.45	1.3471±0.29
PE (18:3_16:0)	GP	PE	35.7731±3.62	34.7099±3.75
PE (16:1_18:2)	GP	PE	1.6181±0.42	2.5024±0.23
PE (16:0_18:1)	GP	PE	41.0052±2.80	58.0738±3.95
PE (18:3_18:1)	GP	PE	3.3569±1.03	2.7347±1.12
PE (18:2_18:3)	GP	PE	38.5134±3.08	40.0714±3.92
PE (18:3_18:3)	GP	PE	2.2047±0.19	2.2192±0.25

PE (22:6_18:2)	GP	PE	0.9941±0.30	1.9630±0.20
PE (18:1_18:2)	GP	PE	40.1059±1.98	66.1324±9.32
PE (17:1_18:2)	GP	PE	2.0355±0.31	2.3373±1.20
PE (16:1_16:0)	GP	PE	1.7230±0.04	1.6755±0.40
PE (18:2_18:2)	GP	PE	382.7631±17.06	431.8563±23.66
PE (18:2_24:0)	GP	PE	2.0920±0.08	2.3772±0.16
PE (20:0_18:2)	GP	PE	1.5708±0.29	1.8653±0.09
PE (18:0_18:2)	GP	PE	41.3607±5.17	45.9946±2.51
PE (18:1_18:1)	GP	PE	5.8158±0.28	19.7984±3.14
PE (17:0_18:2)	GP	PE	1.5445±0.09	1.5863±0.29
PE (18:2_16:0)	GP	PE	449.2160±3.44	488.2067±35.65
PE (16:0_17:2)	GP	PE	3.8297±0.21	3.6812±0.15
PE (18:2_14:0)	GP	PE	7.3340±0.35	7.2448±0.54
PE (18:1_18:0)	GP	PE	3.2645±0.36	3.7547±0.38
PE (18:2_22:0)	GP	PE	5.0483±0.18	5.4837±0.05
PE (18:0_18:3)	GP	PE	3.2421±0.32	3.0479±0.11
PG (18:2_16:0)	GP	PG	150.4454±7.16	135.5291±13.52
PG (18:0_18:1)	GP	PG	0.8387±0.20	0.8474±0.15
PG (16:0_17:0)	GP	PG	0.1805±0.06	N/A
PG (16:0_16:0)	GP	PG	8.5497±0.46	7.8898±0.65
PG (17:1_18:1)	GP	PG	0.8237±0.10	0.6465±0.11
PG (18:0_16:0)	GP	PG	2.2486±0.15	2.0022±0.24
PG (14:0_16:0)	GP	PG	0.7274±0.14	0.6136±0.06
PG (18:1_18:1)	GP	PG	1.8715±0.13	4.5859±0.75
PG (18:2_18:2)	GP	PG	12.9345±0.82	13.1990±2.07
PG (14:0_18:3)	GP	PG	1.1094±0.11	0.8135±0.21
PG (18:0_18:2)	GP	PG	12.1271±0.64	10.5222±2.20
PG (18:3_18:4)	GP	PG	0.3422±0.07	0.2687±0.08
PG (18:1_20:5)	GP	PG	N/A	0.3540±0.15
PG (18:3_18:3)	GP	PG	0.3191±0.03	0.2409±0.13
PG (16:0_18:1)	GP	PG	15.9370±0.49	19.6549±4.40
PG (18:2_18:1)	GP	PG	5.5965±0.93	8.9461±1.09
PG (18:3_16:0)	GP	PG	6.1619±1.05	4.8948±0.34
PG (16:0_16:1)	GP	PG	0.6441±0.05	0.6106±0.19
PI (17:0_18:2)	GP	PI	15.2561±1.86	10.8022±2.07
PI (16:0_18:0)	GP	PI	14.0147±3.98	17.5140±1.09
PI (16:0_18:1)	GP	PI	188.3470±33.67	233.7291±18.28
PI (18:0_18:1)	GP	PI	12.4901±1.47	28.2027±5.25
PI (18:2_16:0)	GP	PI	1607.5845±143.98	1387.3121±65.16
PI (16:1_18:1)	GP	PI	N/A	2.4634±0.83
PI (17:1_18:1)	GP	PI	12.9141±2.22	11.2085±1.20
PI (16:0_16:0)	GP	PI	23.1871±8.60	19.0569±8.69
PI (16:0_20:0)	GP	PI	57.4407±11.12	16.9437±2.00
PI (18:1_18:1)	GP	PI	9.6256±0.43	53.1703±4.59

PI (18:1_18:3)	GP	PI	13.8961±1.51	12.3346±1.09
PI (18:0_18:2)	GP	PI	399.5214±15.63	354.3461±12.93
PI (18:3_18:3)	GP	PI	2.3630±0.85	2.3129±0.28
PI (18:2_18:3)	GP	PI	41.4022±4.88	31.5993±0.88
PI (18:1_18:2)	GP	PI	122.1097±18.62	139.0479±12.74
PI (18:3_16:0)	GP	PI	161.6874±13.84	138.1012±7.61
PI (18:2_16:1)	GP	PI	134.9579±19.34	110.8782±8.33
PI (18:2_18:2)	GP	PI	180.1704±13.23	157.1545±7.76
PMeOH(20:2_20:5)	GP	PMeOH	0.6912±0.18	0.5953±0.15
PMeOH(16:0_22:5)	GP	PMeOH	N/A	0.0795±0.02
PMeOH(18:2_18:3)	GP	PMeOH	0.7916±0.11	0.6222±0.14
PMeOH(16:0_18:2)	GP	PMeOH	4.9453±0.48	4.1756±0.56
PMeOH(18:2_18:2)	GP	PMeOH	5.0508±0.52	4.3665±0.77
PS (18:2_16:0)	GP	PS	10.8351±3.73	48.0796±45.00
PS (18:0_17:1)	GP	PS	37.7461±9.95	40.1767±11.19
PS (16:0_16:0)	GP	PS	18.5312±2.20	18.4811±14.04
PS (16:1_18:1)	GP	PS	27.5274±4.56	21.4812±6.98
SPH(d18:0)	SP	SPH	0.0113±0.00	0.0127±0.01
PhytoSph(d18:2)	SP	SPH	0.0340±0.01	0.0318±0.00
SPH(d16:1)	SP	SPH	3.1710±0.35	3.4474±0.52
SQDG (16:0_16:0)	GL	SQDG	0.4408±0.14	0.8150±0.29
SQDG (14:0_16:0)	GL	SQDG	2.5075±0.67	3.4103±1.15
SQDG (18:2_18:3)	GL	SQDG	0.7060±0.25	N/A
SQDG (16:1_18:4)	GL	SQDG	215.3153±35.10	240.7528±39.58
SQDG (18:2_18:2)	GL	SQDG	1.3768±0.10	3.0444±0.29
SQDG (18:1_18:2)	GL	SQDG	0.6450±0.21	7.1398±1.29
SQDG (16:0_18:3)	GL	SQDG	1.2954±0.46	1.6180±1.31
SQDG (16:1_18:2)	GL	SQDG	11.0343±3.09	10.5926±1.84
SQDG (18:1_18:1)	GL	SQDG	0.2600±0.20	9.2286±1.63
SQDG (16:0_18:2)	GL	SQDG	5.9611±1.55	9.6951±1.78
SQDG (16:0_18:1)	GL	SQDG	2.7284±1.08	17.3978±0.67
SQDG (18:1_18:3)	GL	SQDG	N/A	1.1272±0.38
TG (18:3_20:1_20:1)	GL	TG	0.2798±0.01	N/A
TG (17:0_18:1_20:4)	GL	TG	7.3659±0.07	8.7309±0.33
TG (19:1_18:2_18:2)	GL	TG	9.4445±0.34	11.2340±0.51
TG (18:2_18:3_22:0)	GL	TG	0.3872±0.07	0.4539±0.04
TG (16:0_16:1_20:5)	GL	TG	5.0012±0.64	6.0666±0.42
TG (16:0_18:1_22:5)	GL	TG	0.7255±0.13	2.3232±0.32
TG (16:2_18:1_18:3)	GL	TG	2.5764±0.20	3.0006±0.38
TG (16:0_17:1_20:5)	GL	TG	0.5988±0.08	0.6534±0.10
TG (18:1_18:2_18:3)	GL	TG	569.1139±12.35	686.9680±20.28
TG (18:2_18:2_18:2)	GL	TG	853.2353±6.02	N/A
TG (17:2_19:2_19:2)	GL	TG	0.4091±0.07	0.5347±0.03
TG (18:3_18:3_20:0)	GL	TG	0.0407±0.01	0.0423±0.02

TG (16:0_18:3_18:3)	GL	TG	10.1519±0.60	11.8392±1.03
TG (18:1_18:1_18:3)	GL	TG	1822.1952±38.11	N/A
TG (15:0_18:2_18:3)	GL	TG	3.9797±0.50	5.1908±0.08
TG (18:0_18:2_18:3)	GL	TG	1229.2813±44.01	1491.5553±101.62
TG (14:0_18:1_18:4)	GL	TG	0.9037±0.22	1.3668±0.14
TG (18:1_18:3_20:2)	GL	TG	1.9193±0.02	2.4534±0.40
TG (15:1_18:2_18:2)	GL	TG	5.4838±0.33	7.3063±0.43
TG (13:0_16:0_22:5)	GL	TG	0.3182±0.06	0.3114±0.06
TG (16:1_18:2_18:2)	GL	TG	113.7087±5.34	135.6924±11.47
TG (14:0_16:0_22:5)	GL	TG	4.7127±0.09	5.8884±0.41
TG (18:0_18:1_18:4)	GL	TG	N/A	4.4332±0.26
TG (16:3_18:1_18:1)	GL	TG	10.3011±0.85	12.1443±2.54
TG (16:0_17:1_20:4)	GL	TG	2.5038±0.05	3.1697±0.33
TG (17:1_18:2_18:2)	GL	TG	25.6810±0.99	32.6416±2.54
TG (17:1_18:1_18:3)	GL	TG	17.0413±0.14	23.1978±1.63
TG (17:1_17:2_19:2)	GL	TG	0.1805±0.03	0.1569±0.13
TG (17:0_18:2_18:3)	GL	TG	1.1126±0.06	1.3169±0.32
TG (18:1_18:2_18:2)	GL	TG	1860.6020±42.03	2080.6514±114.57
TG (14:0_18:1_20:4)	GL	TG	0.1757±0.07	0.2607±0.04
TG (18:2_18:3_20:1)	GL	TG	9.7308±0.31	12.5441±0.91
TG (16:0_20:3_20:5)	GL	TG	9.0154±4.52	13.2627±1.14
TG (16:2_16:3_18:2)	GL	TG	N/A	0.0448±0.01
TG (16:0_16:2_18:3)	GL	TG	0.3215±0.04	0.3777±0.03
TG (14:0_20:5_22:6)	GL	TG	0.2269±0.08	0.2328±0.09
TG (12:0_20:5_22:6)	GL	TG	0.0813±0.01	0.0967±0.05
TG (18:1_20:4_22:5)	GL	TG	5.5321±0.34	6.2270±0.82
TG (18:2_18:2_22:6)	GL	TG	0.2526±0.04	0.4363±0.07
TG (14:0_20:4_22:6)	GL	TG	N/A	0.0655±0.06
TG (16:3_18:2_22:5)	GL	TG	102.3226±12.03	140.1328±12.41
TG (17:1_17:3_21:5)	GL	TG	21.7912±1.31	25.5842±0.34
TG (18:2_18:4_18:4)	GL	TG	9.9276±2.08	27.7697±27.61
TG (18:3_18:3_18:4)	GL	TG	0.1421±0.02	0.2092±0.09
TG (16:4_16:4_18:2)	GL	TG	0.0488±0.00	N/A
TG (18:1_20:2_22:6)	GL	TG	8.6960±0.26	10.2110±0.82
TG (22:1_18:2_22:6)	GL	TG	0.3173±0.05	0.4797±0.05
TG (18:3_18:3_18:3)	GL	TG	0.7507±0.05	0.9390±0.05
TG (16:3_18:1_20:5)	GL	TG	157.7084±29.13	226.3428±56.89
TG (18:2_18:3_18:4)	GL	TG	8.0065±1.29	11.1168±1.85
TG (16:3_18:3_18:3)	GL	TG	0.0313±0.01	N/A
TG (16:0_18:2_18:5)	GL	TG	7.5924±0.68	8.1025±1.52
TG (13:0_20:2_20:5)	GL	TG	0.0973±0.05	0.1015±0.03
TG (16:1_18:1_20:5)	GL	TG	1.1586±0.13	1.1519±0.63
TG (18:1_18:1_20:5)	GL	TG	2.3033±0.39	2.6344±0.35
TG (20:1_18:3_18:3)	GL	TG	0.5627±0.04	0.6353±0.15

TG (18:3_18:3_20:1)	GL	TG	0.8865±0.07	1.1018±0.07
TG (18:2_18:3_22:1)	GL	TG	1.7505±0.18	2.3936±0.18
TG (24:1_18:3_18:3)	GL	TG	0.0511±0.01	0.0557±0.00
TG (16:4_18:1_18:3)	GL	TG	7.6958±0.24	10.4157±0.26
TG (17:2_17:3_19:3)	GL	TG	0.2596±0.06	0.3906±0.01
TG (18:2_18:3_18:3)	GL	TG	14.0751±0.67	16.1698±1.30
TG (18:1_18:3_18:4)	GL	TG	68.4651±7.72	90.5973±4.05
TG (18:1_18:2_20:5)	GL	TG	130.5333±5.61	185.0326±13.60
TG (18:3_18:3_20:2)	GL	TG	0.0436±0.01	0.0865±0.01
TG (14:0_18:2_20:6)	GL	TG	0.5017±0.20	0.5144±0.10
TG (16:0_16:1_18:4)	GL	TG	0.0849±0.06	0.1742±0.06
TG (16:0_18:2_18:2)	GL	TG	2127.9445±81.90	2477.8890±127.83
TG (14:0_18:2_18:3)	GL	TG	7.2470±0.73	9.2085±0.17
TG (14:0_16:1_18:1)	GL	TG	26.9975±1.12	36.8094±2.14
TG (14:1_16:0_18:1)	GL	TG	8.5984±4.46	9.7914±5.28
TG (16:0_16:0_16:2)	GL	TG	0.0627±0.03	N/A
TG (16:0_16:1_17:1)	GL	TG	9.5896±0.35	11.4489±0.39
TG (15:0_16:0_18:2)	GL	TG	5.0273±0.26	6.0113±0.54
TG (13:0_18:1_18:1)	GL	TG	2.2137±0.22	1.9259±0.77
TG (16:0_16:0_17:2)	GL	TG	0.5405±0.14	0.7094±0.08
TG(16:1_16:1_17:0)	GL	TG	0.5799±0.08	0.7532±0.04
TG(16:0_16:1_18:1)	GL	TG	401.0109±15.21	475.5216±25.44
TG(14:0_16:1_20:1)	GL	TG	97.8209±13.50	118.3984±5.27
TG(16:0_16:0_18:2)	GL	TG	607.8558±89.75	737.2652±113.06
TG(16:0_17:1_18:1)	GL	TG	152.6337±7.31	182.1858±4.55
TG(16:0_17:0_18:2)	GL	TG	7.8642±0.18	9.5240±0.33
TG(16:0_18:1_18:1)	GL	TG	1913.1692±458.64	1934.1457±178.51
TG(16:0_18:0_18:2)	GL	TG	161.9411±38.36	189.3857±23.94
TG(16:0_16:0_20:2)	GL	TG	1619.6248±199.95	1883.0120±124.32
TG(17:0_18:1_18:1)	GL	TG	307.2261±16.87	357.7919±3.31
TG(18:0_18:1_18:1)	GL	TG	1228.1151±9.87	1366.1149±75.89
TG(16:0_18:1_20:1)	GL	TG	286.4785±86.12	378.8341±155.65
TG(16:1_18:0_20:1)	GL	TG	414.7755±14.00	441.4082±214.61
TG(16:0_16:1_22:1)	GL	TG	1.3798±0.21	1.7659±0.06
TG(16:0_18:2_20:0)	GL	TG	8.2471±0.63	10.3281±1.02
TG(18:0_18:0_18:2)	GL	TG	1698.7645±62.45	1874.7775±143.69
TG(16:0_21:0_18:2)	GL	TG	0.2940±0.04	0.3995±0.08
TG(28:0_18:1_18:1)	GL	TG	0.6299±0.23	0.9030±0.34
TG(14:0_16:0_18:2)	GL	TG	13.8348±0.88	16.5828±1.76
TG(10:0_16:0_18:3)	GL	TG	0.0455±0.02	0.0620±0.01
TG(12:0_16:1_18:1)	GL	TG	16.1852±0.99	18.4757±0.34
TG(12:0_14:0_18:2)	GL	TG	0.0286±0.03	0.0866±0.04
TG(12:0_14:0_16:0)	GL	TG	0.4412±0.15	0.3908±0.06
TG(14:0_14:0_16:0)	GL	TG	0.6138±0.11	0.4402±0.09

TG(14:0_15:0_16:0)	GL	TG	0.2879±0.01	0.3887±0.07
TG(14:0_16:0_16:0)	GL	TG	3.1294±0.44	1.0327±0.21
TG(15:0_16:0_16:0)	GL	TG	0.6269±0.08	0.6419±0.03
TG(16:0_16:0_16:0)	GL	TG	21.9208±1.09	14.7611±0.40
TG(14:0_16:0_18:0)	GL	TG	3.3136±0.25	1.1808±0.41
TG(16:0_18:0_19:0)	GL	TG	0.2627±0.01	0.2554±0.05
TG(16:0_18:0_23:0)	GL	TG	0.0936±0.03	0.1390±0.02
TG(12:0_12:0_16:1)	GL	TG	0.1554±0.03	0.1144±0.05
TG(12:0_16:0_16:1)	GL	TG	0.2441±0.02	0.3845±0.17
TG(14:0_14:0_16:1)	GL	TG	0.0727±0.05	0.1533±0.05
TG(14:0_15:0_16:1)	GL	TG	0.1083±0.09	0.3024±0.11
TG(12:0_16:0_18:1)	GL	TG	2.8270±0.11	3.3276±0.38
TG(14:0_16:0_16:1)	GL	TG	0.2954±0.03	0.9000±0.22
TG(14:0_14:0_18:1)	GL	TG	0.4745±0.06	0.7643±0.12
TG(15:0_16:0_16:1)	GL	TG	0.4785±0.07	0.8614±0.33
TG(16:0_16:0_16:1)	GL	TG	18.2694±1.40	21.2415±2.71
TG(14:0_16:0_18:1)	GL	TG	16.7705±1.79	20.8207±0.33
TG(15:0_16:0_18:1)	GL	TG	7.8037±0.26	10.1572±0.45
TG(16:0_16:0_17:1)	GL	TG	8.7132±0.15	10.8530±0.38
TG(16:0_16:1_17:0)	GL	TG	0.5183±0.02	0.5790±0.03
TG(16:0_16:0_18:1)	GL	TG	815.3466±5.56	955.3973±27.70
TG(16:0_17:0_18:1)	GL	TG	47.9530±1.93	60.3516±1.37
TG(16:0_16:1_20:0)	GL	TG	0.3019±0.04	0.3408±0.01
TG(12:0_16:0_18:2)	GL	TG	0.8246±0.17	1.0873±0.17
TG(14:1_18:2_18:2)	GL	TG	7.6733±2.02	11.0936±0.49
TG(10:0_18:1_18:2)	GL	TG	1.2070±0.31	1.6298±0.11
TG(14:0_16:0_16:3)	GL	TG	0.2207±0.05	0.3302±0.05
TG(16:1_16:1_16:2)	GL	TG	0.0529±0.02	0.0692±0.02
TG(12:0_18:2_18:2)	GL	TG	0.9562±0.15	1.2819±0.32
TG(16:0_16:1_16:3)	GL	TG	0.3142±0.09	0.3396±0.04
TG(15:1_17:1_17:2)	GL	TG	N/A	0.1022±0.08
TG(14:0_18:2_18:2)	GL	TG	29.9580±2.69	38.2573±2.45
TG(16:1_16:1_18:2)	GL	TG	4.3539±0.45	4.8450±0.44
TG(16:0_16:1_18:3)	GL	TG	4.7652±0.29	5.6228±0.45
TG(14:0_18:1_18:3)	GL	TG	16.7923±1.66	21.2803±0.56
TG(16:1_16:2_18:1)	GL	TG	2.2492±0.24	2.8065±0.30
TG(15:0_18:2_18:2)	GL	TG	11.0657±0.47	14.1732±1.07
TG(16:0_17:1_18:3)	GL	TG	2.0596±0.23	2.6428±0.19
TG(16:0_18:1_18:3)	GL	TG	459.9483±20.26	572.4726±32.79
TG(17:1_18:1_18:2)	GL	TG	100.0993±3.03	123.8973±3.09
TG(16:0_16:0_21:4)	GL	TG	0.7659±0.08	0.9226±0.04
TG(17:0_18:2_18:2)	GL	TG	66.5384±0.97	77.8645±1.75
TG(18:1_18:1_18:2)	GL	TG	2096.1739±31.60	2243.6927±103.68
TG(16:0_18:2_20:2)	GL	TG	38.3832±4.22	45.6947±2.63

TG(16:0_18:1_20:3)	GL	TG	N/A	1.3331±1.19
TG(18:0_18:1_18:3)	GL	TG	47.2813±5.84	75.1160±32.40
TG(16:0_18:3_22:1)	GL	TG	2.0371±0.07	2.4186±0.11
TG(16:0_20:2_20:2)	GL	TG	13.2685±1.07	16.5439±1.33
TG(18:0_18:2_20:2)	GL	TG	1.0373±0.17	1.3683±0.26
TG(18:0_18:3_20:1)	GL	TG	203.4974±4.29	234.1235±21.22
TG(18:1_18:1_20:2)	GL	TG	87.0801±10.46	88.3080±11.17
TG(12:0_16:3_18:2)	GL	TG	0.0423±0.01	0.0606±0.00
TG(10:0_18:2_18:2)	GL	TG	0.3235±0.06	0.4000±0.04
TG(14:0_14:0_18:3)	GL	TG	N/A	0.0297±0.01
TG(21:0_18:1_18:2)	GL	TG	0.7265±1.04	1.8569±1.54
TG(18:1_18:2_20:0)	GL	TG	37.7202±9.87	45.4673±1.60
TG(13:0_16:0_18:3)	GL	TG	N/A	0.0381±0.01
TG(14:0_16:1_18:2)	GL	TG	1.8432±0.44	2.3664±0.21
TG(16:0_16:1_16:2)	GL	TG	0.1364±0.02	0.1144±0.04
TG(13:0_18:1_18:2)	GL	TG	0.3645±0.01	0.3856±0.07
TG(16:0_15:1_18:2)	GL	TG	2.0676±0.10	2.6783±0.11
TG(16:1_16:1_18:1)	GL	TG	41.2202±2.68	49.3042±3.12
TG(16:0_16:1_18:2)	GL	TG	144.4925±7.44	175.8629±5.52
TG(16:0_16:2_18:1)	GL	TG	2.4502±0.46	2.9491±0.61
TG(16:0_16:0_18:3)	GL	TG	23.9882±6.68	32.2079±1.94
TG(14:0_18:1_18:2)	GL	TG	73.4770±12.34	114.9723±6.29
TG(15:0_18:1_18:2)	GL	TG	21.1480±0.93	27.0611±1.52
TG(16:0_17:1_18:2)	GL	TG	25.8793±1.43	32.1417±1.27
TG(16:1_16:2_19:0)	GL	TG	0.0672±0.02	0.0949±0.01
TG(16:0_17:0_18:3)	GL	TG	0.8011±0.09	1.0159±0.08
TG(16:0_18:1_18:2)	GL	TG	1724.8501±180.62	2074.6152±253.39
TG(16:1_18:1_18:1)	GL	TG	578.3034±12.37	703.6556±13.97
TG(14:0_18:1_20:2)	GL	TG	5.2085±1.04	5.6499±0.56
TG(16:0_18:0_18:3)	GL	TG	16.6017±0.82	20.4238±1.34
TG(17:0_18:1_18:2)	GL	TG	66.1442±3.76	79.1863±1.36
TG(18:1_18:1_18:1)	GL	TG	977.8799±76.21	1071.6721±44.86
TG(18:0_18:1_18:2)	GL	TG	703.5037±26.41	824.2897±37.34
TG(16:0_18:1_20:2)	GL	TG	67.2507±3.99	79.3283±2.17
TG(16:0_18:2_20:1)	GL	TG	62.9785±4.52	77.0209±1.89
TG(20:0_18:1_18:2)	GL	TG	0.4577±0.16	0.4629±0.15
TG(16:0_18:3_20:0)	GL	TG	0.9229±0.11	0.8984±0.20
TG(18:1_18:1_20:1)	GL	TG	1997.2694±133.45	2087.1236±160.73
TG(16:0_16:1_16:1)	GL	TG	22.3191±5.95	31.1086±4.52

Note: Value reported is mean ± standard deviation (n = 3). N/A means not detected.
